# Commitments increase preparedness for floods

**DOI:** 10.1371/journal.pone.0219993

**Published:** 2019-08-15

**Authors:** Piers D. L. Howe, Adriana Vargas-Sáenz, Ilona M. McNeill

**Affiliations:** School of Psychological Sciences, University of Melbourne, Melbourne, Victoria, Australia; Qazvin University of Medical Sciences, ISLAMIC REPUBLIC OF IRAN

## Abstract

The current study investigated whether we could encourage Australian residents to become better prepared for floods by inviting them to make a specific commitment to do so. We sampled 374 residents of the state of Victoria (56% male, 81% metropolitan) and 400 residents of the state of New South Wales (45% male, 59% metropolitan) who lived in locations that were potentially at risk of floods. They residents were sampled so that their distributions of ages, genders and living locations were as representative as possible of the population of those two states. These residents completed two surveys that ascertained their preparedness for floods at two points in time, separate by a two-week period. At the end of the first survey all residents received information about how they could better prepare for floods. In addition, approximately half the residents were randomly selected to be invited to commit to becoming better prepared for floods. We found that 74% of residents who were invited to commit to becoming better prepared for floods, were willing to make this commitment. We found that the group that was invited to commit to become better prepared for floods increased their preparedness for floods over the two-week period that separated the two surveys more than the group that was not invited to make this commitment, *F*(1, 772) = 4.53, *p* = .034, η^2^ = .006. We conclude that when emergency services inform residents of flood-prone areas how to better prepare for floods, they should also attempt to elicit from the residents a commitment to become better prepared for floods.

## Introduction

Between 1900 and 2015, at least 1859 people were killed by floods in Australia [1], making floods historically the second most deadly natural disaster after heatwaves [2]. Floods are also the single most costly natural disaster. For instance, from 1967 to 1999 floods cost Australia AU$10.4B (calculated in 1999 Australian dollars), which corresponds to 29% of the total natural disaster costs for this period [3]. Better preparedness for floods can help reduce the socioeconomic impact caused by these types of events (e.g., number of lives lost, damage to property, etc.). Not surprisingly, local governments and emergency services put considerable efforts and resources into encouraging people to become better prepared for floods.

The Australian state emergency services periodically run information campaigns to encourage Australian residents to become better prepared for floods. These campaigns inform residents that they can help prepare themselves for floods by performing the following four actions: checking that their home or contents insurance covers flood damage, keeping a list of emergency telephone numbers by their phone (or storing a list on their mobile phone), preparing an emergency plan, and assembling an emergency kit [4]. However, a previous study on flood preparedness amongst Australian residents of flood-prone areas found that less than 50% could confirm that they were properly insured against floods and, for each of the actions needed to form an emergency plan, less than one in three respondents reported having completed the action (McNeill, Boldero, & Vargas-Sáenz, in press). This raises the question of how prepared residents of flood-prone areas are for floods and how best to increase their preparedness.

Previous research has shown that having people commit to performing an action increases the chance of them performing that action [5, 6]. For example, Milkman et al. [7] found that prompting employees to commit, in writing, to being vaccinated against influenza at a particular time and date increased the number of employees that were vaccinated by 13% relative to when they were not prompted to make a commitment. Similarly, Martin et al. [8] found that having patients write down the time and date of their next doctor’s appointment reduced the number of missed appointments by 18%, relative to their non-treatment control condition. Based on these findings, having people commit to a particular action would seem to be an effective way of modifying behaviour.

Such a hypothesis follows naturally from the theory of planned behaviour [9, 10]. According to this theory, attitude, perceived social norms and perceived behavioural control give rise to intention, which in turn gives rise to behaviour. Since, intentions directly determine behaviour, it follows that altering the intentions should alter the behaviour. As commitments are a natural way to alter intentions, it is unsurprising that commitments should affect behaviour. Based on both this theory [9, 10] and the previous studies on the effect of commitment on behaviour [7, 8] we expected that requesting that participants make a specific commitment to perform a particular action should be a highly effective way of altering their behaviour. The purpose of our study was to test whether this prediction would hold in the context of flood preparedness. Specifically, we wished to investigate whether inviting residents of flood-prone areas to commitment to becoming better prepared for floods would result in them increasing their preparedness for floods more than residents who had not been invited to make these commitments.

## Methods

### Participants

Our participants were recruited using a survey company called the Online Research Unit (ORU). The ORU is an Australian survey company and is certified by the International Organization for Standardization (ISO 20252 and ISO 26362). The ORU maintains a panel of volunteers who have agreed to participate in online surveys. A mix of incentives including vouchers and charitable donations of small value are provided to participants via the ORU in return for their survey participation. All participants gave informed consent and our study was approved by the University of Melbourne, School of Psychological Sciences, Human Ethics Advisory Group (Id: 1750302). To be included in our survey, participants needed to be at least 18 years old, self-identify as living in a property that could be potentially flooded if the weather conditions were extreme enough, and self-identify as someone who would either make the decisions or at least share the decision-making around flood safety. The survey company recruited participants so that their distributions of ages, genders and living locations were as representative as possible of the populations of the states of Victoria and New South Wales. Because we were attempting to fit simultaneously to three separate distributions, the distribution of our participants did not quite match those of the broader population, as detailed in S1 Appendix.

### Procedure and design

Participants were surveyed in two waves. In Wave 1, participants were randomly allocated either to the Standard condition or to the Commitment condition. In both conditions, participants were asked to complete an online survey. As this survey included items for an unrelated study, here we will report on only those aspects of the survey that are relevant to the current study. In the Standard condition, participants were asked whether they had checked that their house or home contents insurance policy covers flooding and whether they had a list of emergency contact numbers either near or in their phone. They were then asked to indicate which of 12 specific natural disaster preparedness actions they had performed, in constructing their home emergency plan. Finally, they were asked to indicate which of 11 specific items they had either placed in their emergency kit or had ready to be immediately placed in their home emergency kit, should they be threatened by a flood. The 12 specific actions and the 11 specific items were based on what our end-users required emergency plans and emergency kits to contain, as described in McNeil et al. [11]. Participants were then asked a series of four questions designed to determine to what degree they believed that they could become better prepared for floods, should they choose to do so. The specific actions, items and questions are listed in the S2 Appendix.

The survey used in the Commitment condition was identical to the survey used in the Standard condition, except that after ascertaining to what degree participants believed they could better prepare for floods, should they do so, the participants were then asked to commit to becoming better prepared for floods. This was done by first asking if they were, in principle, interested in improving their household’s preparedness for floods. Those who replied in the affirmative were then asked to choose at least one action that would increase their preparedness for floods. They were then thanked for making the commitment and asked to place in their diary a specific time and date to perform this action. Finally, they were asked to confirm that they had placed this reminder in their diary. At the end of both conditions, participants were informed that we would survey them again in approximately two weeks’ time and provided a link to a webpage that provided information as to how they could better prepare for floods. The transcript for the commitment questions and the information webpage is included in S2 Appendix.

Two weeks after receiving the Wave 1 survey, participants in both the Standard condition and the Commitment condition received the second survey. This survey assessed their preparedness using the same questions as the previous survey. The only exception was for participants in the Commitment condition: those who in Wave 2 reported not having performed the action(s) they had previously committed to performing in Wave 1 were then asked the reason why they had not done so.

### Analysis

In analyzing our results, we considered the four categories of actions that our surveys encouraged the participants to focus on. These four categories were: checking whether their household insurance covered flooding, looking up the relevant emergency phone numbers and placing them near or in their phones, preparing a home emergency plan for floods and assembling an emergency kit. For each category, we determined whether their preparedness in Wave 2 had increased, decreased or stayed the same relative to their preparedness in Wave 1, scoring these three possibilities as +1, -1 and 0 respectively. For example, if, in Wave 2, a participant reported completing more planning actions than they had reported having completed in Wave 1, they would receive a score of +1 for this category. Conversely, if they reported having fewer emergency kit items in Wave 2 than they reported having in Wave 1, they would receive a score of -1 for this category.

We first calculated the increase in overall preparedness in Wave 2 relative to Wave 2, which we defined to be the sum of the increase in preparedness across all four categories. We ran an independent samples ANOVA to compare the overall increase in preparedness in the Commitment condition relative to the Standard condition. We then ran a series of chi-squared tests to analyse the increase in preparedness in each of the four categories in the Commitment condition relative to the Standard condition in Wave 2 relative to Wave 1. We defined statistical significance as corresponding to p < .05.

## Results

The raw data can be found in S1 Data. For the participants who received both the Wave 1 and Wave 2 surveys (*n* = 2011), we excluded anyone who failed to answer correctly two out of three randomly placed attention checks (*n* = 376) and those who partially completed either survey (*n* = 861). These exclusions resulted in a final sample of 403 and 371 participants for the Standard and Commitment conditions, respectively.

In Wave 1, only 1.4% of residents reported being completely prepared for floods. The reported preparedness of each of the four components was as follows: 72.5% reported having already checked their insurance, 56.5% reported having already looked up all relevant emergency contact numbers, 22.4% reported having already obtained all 11 items for their emergency kit, and only 1.9% reported having already completed all 12 planning actions.

In Wave 1, for the Commitment condition, 81.9% agreed that, in principle, they wished to become better prepared for floods. Of these, 89.9% committed to performing at least one action. 46.9% committed to making sure that their home and contents insurance covers flooding, 52.4% committed to looking up relevant emergency telephone numbers and placing them near their phone, 40.7% committed to preparing or improving their emergency plan, and 46.2% committed to preparing or improving their emergency kit. Of those who had committed to performing at least one action, 57.5% said they had actually placed a reminder in their diary to do so.

Averaging across the two conditions, the perceived control was high. On a scale that ran from 4 to 28, with higher values indicating greater degrees of perceived control, participants averaged 20.9, with a standard deviation of 4.5. This shows that participants believed that that they had the ability to become better prepared for floods, had they chosen to do so.

### Overall preparedness

We defined a person’s increase in overall preparedness as the sum of the increase in preparedness over each of the four categories. The mean increase in overall preparedness from Wave 1 to Wave 2 was 0.30 (SD = 1.50) for the Standard condition and 0.54 (SD = 1.66) for the Commitment condition. The difference between the two conditions was statistically significant *F*(1, 772) = 4.53, *p* = .034, η^2^ = .006.

#### Individual analyses

Since the increase in the overall preparedness was significantly greater in the Commitment condition in comparison to the Standard condition, we analysed each of the four components in turn to determine for which of these components a similar finding held. So as to have an overall alpha of .05, we used a Bonferroni correction and performed each of the four tests at the .0125 significance level. We found that only for the Planning component was the increase in preparedness in Wave 2 relative to Wave 1 significantly greater in the Commitment condition than in the Standard Condition.

**Insurance.** For this component, in the Standard condition, 29 participants had increased their preparedness, 72 had decreased their preparedness and the remaining 302 had not changed their preparedness in Wave 2 relative to Wave 1. Conversely, in the Commitment condition, 35 had increased their preparedness, 62 has decreased their preparedness and the remaining 274 had not changed their preparedness. The difference between the two conditions was not statistically significant, Pearson *X*^*2*^ (1, *n* = 774) = .034, *p* = .34.

**Emergency contact phone numbers.** For this component, in the Standard condition, 62 participants had increased their preparedness, 66 had decreased their preparedness and the remaining 275 had not changed their preparedness in Wave 2 relative to Wave 1. Conversely, in the Commitment condition, 79 had increased their preparedness, 54 has decreased their preparedness and the remaining 238 had not changed their preparedness. The difference between the two conditions was not statistically significant, Pearson *X*^*2*^ (1, *n* = 774) = .067, *p* = .064.

**Planning.** For this component, in the Standard condition, 146 had increased their preparedness, 121 had decreased their preparedness and the remaining 136 had not changed their preparedness in Wave 2 relative to Wave 1. Conversely, in the Commitment condition, 168 had increased their preparedness, 83 has decreased their preparedness and the remaining 120 had not changed their preparedness. The difference between the two conditions was statistically significant, Pearson *X*^*2*^ (1, *n* = 774) = .104, *p* = .004.

**Emergency kit.** For this component, in the Standard condition, 213 had increased their preparedness, 69 had decreased their preparedness and the remaining 121 had not changed their preparedness in Wave 2 relative to Wave 1. Conversely, in the Commitment condition, 196 had increased their preparedness, 77 has decreased their preparedness and the remaining 98 had not changed their preparedness. The difference between the two conditions was not statistically significant, Pearson *X*^*2*^ (1, *n* = 774) = -.024, *p* = .513.

## Discussion

We found that the increase in overall preparedness in Wave 2 relative to Wave 1 was 0.30 (SD = 1.50) for the Standard condition and 0.54 (SD = 1.66) for the Commitment condition. Thus, it was 80% larger in the Commitment condition than in the Standard condition. Considering each of the four components in turn, only for the Planning component was the increase in preparedness in Wave 2 relative to Wave 1 significantly greater in the Commitment condition than in the Standard condition. So, while our results were consistent with our expectations that commitments would lead to an increase in overall preparedness, a more nuanced analysis found evidence only that commitments increased planning preparedness.

Our results would seem to be broadly consistent with the previous literature in that we found that commitments can modify behaviour. However, the percentage increase found in our study was much larger than the percentage increase reported by previous studies. For example, Milkman et al. [7] found that having employees commit, in writing, to being vaccinated at a particular time and date increased the vaccination rate by 13% relative to their no-treatment control group. Similarly, Martin et al. [8] they found that having patients write down the time and date of their next appointment reduced the number of missed appointments by 18% relative to their no-treatment control group.

Given that our effect size when presented as a percentage seems to be very large, it is surprising that it is so small when represented as a partial eta squared value. One reason for this is that partial eta squared represents the variance accounted for by the treatment relative to the total variance in the sample. In other words, it is a (scaled) measure of the absolute difference between the two conditions, not the percentage difference between the two conditions. Despite the Commitment condition representing a large percentage improvement in overall preparedness over the Standard condition, the absolute difference in the increase in overall preparedness in the Commitment condition relative to the Standard condition was quite small, just 0.24 points out of a maximum of 4 points.

Why then was the absolute difference between the two conditions so small? A priori, one possibility is that this could reflect a ceiling effect. It could be that all residents were already so well prepared for floods that it was not possible for them to become better prepared. However, this possibility does not seem to be supported by our data. Only 1.4% of participants reported in Wave 1 that they were completely prepared and that there was nothing further they could do to prepare for floods. As such, there seems to have been plenty of scope for residents to have become better prepared.

All the participants who acknowledged not performing the action(s) that they agreed to perform were asked why they had not done so. While 30% reported that they had not had enough time to perform the action(s), which may point to a lack of motivation, 63% reported that they had simply forgotten to do so. This may have in part been due to the relative long interval between the two surveys (two weeks) and the fact that only 58% of participants in the Commitment condition confirmed that they had actually placed a reminder in their diary to perform their designated preparedness action.

It appears that the main reason why participants failed to perform the action(s) that they had committed to doing was that they simply forgot to do so. Future work will need to investigate to what extent preparedness can be increased by periodic reminders. In particular, it will need to be established whether periodically reminding people of the commitments that they have made will increase preparedness further. Knowing the best way to encourage people to increase their own preparedness has significant implications and has the potential to save lives.

### Limitations

One limitation with our study was that we relied on participants self-reporting their degree of preparedness. We could not and did not independently verify these self-reports. It is possible that reports were biased with participants either under or over-reporting their preparedness. However, there is no reason to suspect the bias would be different in the two conditions. Thus, our paradigm still allows us to determine whether our intervention was successful.

A possible reason why our Commitment condition was not more effective was that our participants may have perceived that they lacked the ability to prepare for floods. According to the theory of planned behaviour intention should give rise to action, but this can be modified by the extent to which participants believe that they can cause the action to occur [9, 10]. Lack of perceived ill control will decrease intention and will also decrease attempts to take action even when the intention to perform the action is present. It could be that people felt that they lacked the ability to become better prepared for floods, to the extent that they did not even attempt to increase their preparedness. In particular it could be that some participants could not afford to become better prepared for floods. As we did not ask about income in our demographics questionnaire we cannot address this concern directly. However, this possibility was not consistent with our finding that, on a scale from 4 to 28, the average perceived control was 20.9. This shows that participants reported high perceived control and felt that they could have become better prepared for floods had they chosen to do so.

Another possible limitation is that the demographics of our participants did not precisely match those of the Victorian and New South Wales populations in general. Because we tried to simultaneously match on three dimensions, location, gender and age, we did not perfectly match on any single dimension. Additionally, our study relied on our participants self-identifying as being at risk of floods. This may have biased our sample to include participants who were more aware of their flood risk than normal. As such, we cannot say that our study is completely representative of the population in general. However, we are still able to say that our data indicates that, in general, commitments increase preparedness as the participants were randomly allocated to the two conditions in our study.

## Conclusions

In Australia, floods are the most costly and the second most deadly natural disaster. It is therefore imperative that residents of flood-prone areas adequately prepare for floods. Currently, local governments and emergency services encourage residents to prepare for floods by telling them what they should do. Here we found that inviting residents to commit to preparing for floods lead to a greater increase in overall preparedness. However, our intervention was not as effective as it could have been because many residents reported forgetting to perform the action(s) that they had committed to performing. At this stage, it is unclear to what extent the barrier to action is forgetfulness, not a lack of commitment. Future work will need to address this issue so we can better design interventions to increase overall flood preparedness.

## Supporting information

S1 Appendix Demographics. (DOCX)

S2 Appendix Survey. (DOCX)

S1 Data Anonymised raw data. (SAV)
