# Supplementary material for: Commitments increase preparedness for floods
Source: PLoS One. 2019 Aug 15;14(8):e0219993. doi: 10.1371/journal.pone.0219993 (PMC6695169; doi:10.1371/journal.pone.0219993)
Supplement: S1 Appendix — (DOCX) [file pone.0219993.s001.docx]

**S1 Appendix - Demographics**

|  |  |  | |  |  |  |  | |
| --- | --- | --- | --- | --- | --- | --- | --- | --- |
| **W1 VICTORIA (Commitment condition)** | | | |  | **W1 NSW (Commitment condition)** | | | |
| No. of valid cases | 177 | | |  | No. of valid cases | 194 | | |
|  |  |  | |  |  |  |  | |
| **LOCATION** | **ABS %** | | **DATA %** |  | **LOCATION** | **ABS %** | | **DATA %** |
| Melbourne | 76.47% | | 81.40% |  | Sydney | 64.99% | | 59.30% |
| Rest of VIC | 23.53% | | 18.60% |  | Rest of NSW | 35.01% | | 40.70% |
|  |  | |  |  |  |  | |  |
| **GENDER** | **ABS %** | | **DATA %** |  | **GENDER** | **ABS %** | | **DATA %** |
| Male | 49.48% | | 55.90% |  | Male | 49.56% | | 45.40% |
| Female | 50.52% | | 44.10% |  | Female | 50.44% | | 54.60% |
|  |  | |  |  |  |  | |  |
| **AGE** | **ABS %** | | **DATA %** |  | **AGE** | **ABS %** | | **DATA %** |
| 18-24 | 12.63% | | 4.00% |  | 18-24 | 12.00% | | 4.60% |
| 25–29 | 10.10% | | 7.79% |  | 25–29 | 9.64% | | 6.70% |
| 30–34 | 9.97% | | 10.70% |  | 30–34 | 9.53% | | 6.20% |
| 35–39 | 8.84% | | 12.00% |  | 35–39 | 8.69% | | 10.80% |
| 40–44 | 8.36% | | 9.00% |  | 40–44 | 8.34% | | 7.70% |
| 45–49 | 8.55% | | 9.60% |  | 45–49 | 8.40% | | 9.30% |
| 50–54 | 7.86% | | 12.40% |  | 50–54 | 7.96% | | 9.80% |
| 55–59 | 7.58% | | 11.90% |  | 55–59 | 7.93% | | 12.90% |
| 60–64 | 6.71% | | 5.10% |  | 60–64 | 7.05% | | 11.90% |
| 65–69 | 5.99% | | 10.20% |  | 65–69 | 6.30% | | 9.30% |
| 70–plus | 13.41% | | 6.20% |  | 70–plus | 14.16% | | 10.80% |
|  |  |  | |  |  |  |  | |
| **W1 VICTORIA (Standard only)** | | | |  | **W1 NSW (Standard only)** | | | |
| No. of valid cases | 197 | | |  | No. of valid cases | 206 | | |
|  |  |  | |  |  |  |  | |
| **LOCATION** | **ABS %** | | **DATA %** |  | **LOCATION** | **ABS %** | | **DATA %** |
| Melbourne | 76.47% | | 81.70% |  | Sydney | 64.99% | | 58.70% |
| Rest of VIC | 23.53% | | 18.30% |  | Rest of NSW | 35.01% | | 41.30% |
|  |  | |  |  |  |  | |  |
| **GENDER** | **ABS %** | | **DATA %** |  | **GENDER** | **ABS %** | | **DATA %** |
| Male | 49.48% | | 49.20% |  | Male | 49.56% | | 50.50% |
| Female | 50.52% | | 50.80% |  | Female | 50.44% | | 49.50% |
|  |  | |  |  |  |  | |  |
| **AGE** | **ABS %** | | **DATA %** |  | **AGE** | **ABS %** | | **DATA %** |
| 18-24 | 12.63% | | 5.6% |  | 18-24 | 12.00% | | 6.80% |
| 25–29 | 10.10% | | 8.6% |  | 25–29 | 9.64% | | 6.30% |
| 30–34 | 9.97% | | 10.7% |  | 30–34 | 9.53% | | 10.20% |
| 35–39 | 8.84% | | 9.1% |  | 35–39 | 8.69% | | 11.20% |
| 40–44 | 8.36% | | 10.2% |  | 40–44 | 8.34% | | 6.80% |
| 45–49 | 8.55% | | 6.6% |  | 45–49 | 8.40% | | 10.20% |
| 50–54 | 7.86% | | 10.7% |  | 50–54 | 7.96% | | 11.20% |
| 55–59 | 7.58% | | 9.6% |  | 55–59 | 7.93% | | 7.80% |
| 60–64 | 6.71% | | 8.1% |  | 60–64 | 7.05% | | 12.60% |
| 65–69 | 5.99% | | 8.6% |  | 65–69 | 6.30% | | 5.30% |
| 70–plus | 13.41% | | 12.2% |  | 70–plus | 14.16% | | 11.70% |

**Notes**

ABS = Population data obtained from the Australian Bureau of Statistics

Data = Data obtained from our surveys

Gender and age distributions of general population obtained from table 8 Australian Bureau of Statistics, Australian Demographic Statistics, Jun 2017, 31010DO002_201706 downloaded from <http://www.abs.gov.au/AUSSTATS/abs@.nsf/DetailsPage/3101.0Jun%202017?OpenDocument>

on 17 November, 2018.

Location distributions of general population obtained from Australian Bureau of Statistics, Population by Age and Sex, Regions of Australia, 2016 downloaded from

<http://www.abs.gov.au/AUSSTATS/abs@.nsf/DetailsPage/3235.02016?OpenDocument>

on 17 November, 2018.
