# Supplementary material for: Commitments increase preparedness for floods
Source: PLoS One. 2019 Aug 15;14(8):e0219993. doi: 10.1371/journal.pone.0219993 (PMC6695169; doi:10.1371/journal.pone.0219993)
Supplement: S2 Appendix — (DOCX) [file pone.0219993.s002.docx]

**S2 Appendix - Survey**

**Section 1: Preparedness Questions**

*In Wave 1, both the Standard condition and Commitment condition saw the following questions. The Home Emergency Plan measure and the Emergency Kit measure were based on [11].*

**Insurance**

- You have made sure your home insurance or contents insurance covers flooding.

**Emergency Contact Numbers**

- You have looked up relevant emergency phone numbers and placed them near or in your phone.

**Home Emergency Plan**

Participants were asked to indicate which of the following 12 actions they had performed.

- A suitable relocation destination for you and any other household members (including children and pets) has been selected and all adult members of your household have agreed upon this destination.
- You have identified what height floodwater can get to in and around your property (council/historical floods/SES) and at what heights local roads close. You have determined what height this corresponds to on your local flood gauge (river) or what level of local rainfall contributes to (flash) flooding. All adult members of your household are aware of this information.
- All adult members of your household have discussed or considered how different water levels and other reasons for road closures might impact evacuation/relocation plans. You have identified alternative evacuation routes and have determined whether and how such road closures would impact on the triggers for evacuation (e.g., whether certain road closures may mean you need to leave earlier than anticipated).
- All adult members of your household have thought carefully about what (each of) you would need to do in response to a flood, and how this would be affected by different circumstances, including when different householders are at different locations (e.g., one is at home, one is at work, kids at school), or when certain householders may not have access to a vehicle (e.g., due to another householder using it and not being home) or at different times of the day/week (e.g., at night)).
- You (and anyone who might be involved in the plan) have taken into account atypical or unexpected situations (e.g., certain family members not being at home even though they normally are, or friends/family visiting who are unable to evacuate themselves without help), and have agreed with all relevant people on who will do what in such situations.
- All adult members of your household are aware of both the flood warnings system that is relevant for your locality and of natural signs of flooding so that you can respond quickly.
- Specific triggers for starting to undertake preparatory actions and/or leaving (e.g., height on flood gauge, getting a Bureau of Meteorology (BoM) warning on a mobile phone, hearing a warning on radio) have been identified and agreed upon by all adult members of your household.
- All important things to do and remember in case of a flood have been listed (written or typed on computer, phone, etc.).
- Your household has practiced/rehearsed your flood plan at least once over the last 12 months.
- All adult household members have thought about the consequences a flood would have on you and your household in the days, weeks, and months following the flood (e.g., your employment, schooling, etc.), and you have planned for these consequences to minimize their impact. All adult members of your household have agreed upon this plan.
- All people who play a part in the successful execution of your flood plan (e.g., household members, neighbours, people you plan to stay with) are aware of the plan and have agreed to their role in the plan.
- All adult members of your household are aware of your community's flood plan.

**Emergency Kit**

For each of the following 16 items, participants were asked to indicate whether they had placed the item in a designated home emergency kit (**Assembled**), they haven’t placed the item in a designated emergency kit but have it available to be immediately placed in a designated emergency kit (**Available**) or they currently don’t have the item readily available to be placed in an emergency kit (**Unavailable**).

- Portable battery-operated AM/FM radio with spare batteries
- Torch with spare batteries
- A folder with copies of important documents and records (e.g., insurance, passport, prescriptions)
- First aid kit and manual (with medical supplies necessary for your household)
- A waterproof bag/container for your valuables
- Candles and waterproof matches
- An emergency source of lighting and warmth/cooling that could be used in the days following a flood
- Sturdy gloves
- Long life food (3 days supply) including the ability to prepare the food in the days following a flood
- Bottled water (3 days supply at 3 litres per person per day)
- A waterproof bag/container for the above items to be placed in

**Questions evaluating perceived behavioural control**

In this section of the survey, we are interested in mapping differences in household resilience in relation to floods, and in getting a better picture of why some households and communities are more prepared than others.

This next set of questions asks you about your ability to prepare for floods as recommended by the Victorian SES.

For me to carry out the preparedness activities that are recommended for preparing my household for floods would be

Impossible 1 2 3 4 5 6 7 Possible

*If I wanted to I could carry out the preparedness activities that are recommended for preparing my household for floods

Definitely true 1 2 3 4 5 6 7 Definitely untrue

How much control do you believe you have over carrying out the preparedness activities that are recommended for preparing your household for floods?

No control 1 2 3 4 5 6 7 Complete control

*It is mostly up to me whether or not I carry out the preparedness activities that are recommended for preparing my household for floods

Strongly agree 1 2 3 4 5 6 7 Strongly disagree

**These questions were reverse coded*

**Section 2: Additional Questions for the Commitment Condition**

*Only participants in the Commitment condition saw these questions. Participants in the Standard condition skipped this section.*

[Their name], are you, in principle, interested in improving your household’s preparedness for floods?

- Yes, in principle, I would like to become better prepared for floods.
- No, I have no interest in becoming better prepared for floods.

*If they said yes to the above they were then shown:*

[Their name], to become better prepared for floods, can you please commit to performing at least one action on the following list that you have **not**already performed within the next two weeks. (At the end of this survey we will give you links to all the documents and information that you will need to complete these actions).

- Make sure your home and contents insurance covers flooding.
- Look up relevant emergency phone numbers and place them near or in your phone.
- Prepare or improve your Emergency Plan.
- Prepare or improve your Emergency Kit.
- I don’t wish to commit to performing any of these actions.
- I have already performed all these actions and it is not possible to improve either my Emergency Plan or my Emergency Kit.

*If they chose at least one action to perform, they were then shown*

[Their name], thank  you for making that commitment. Please choose a specific time and date to perform this action. Please place this information in your diary or calendar **now.**

If you don’t use a diary or calendar, there are a number of free websites that will send you an email to remind you, e.g. <http://www.memotome.com/>

*They were then shown*

[Their name], please confirm that you have noted the time and date that you will perform this action in your diary or calendar, or otherwise set up a system to remind you to perform this action.

- Yes, I have done this
- No, I haven’t done this yet, but will do so
- No, I won’t do this as I don’t think it is necessary

**Section 3: Conclusion**

*Participants in both the Standard condition and the Commitment conditions.*

Thank you for participating in this survey. We will survey you again in approximately 2 weeks time.

In the meantime, to help you improve your preparedness, please click on and save the following link: [How to plan and prepare for floods](https://melbpsych.co1.qualtrics.com/CP/File.php?F=F_eQVm2zhRE2OW741)

This page contains:

- instructions on how to check your insurance policy,
- information on where to find the phone numbers relevant during a flood emergency,
- a list of aspects that need to be addressed in your flood emergency plan, and
- a list of items to prepare for your emergency kit.

Please click "Next" to finalise the survey session and be redirected to the ORU website.

**Section 4: How to plan and prepare for floods**

*Participants who clicked on the above link were then shown the following information.*

**How to Plan and Prepare for Floods**

In terms of dollars and lives lost, floods cause more damage per year than any other natural hazard in Australia. To limit the tremendous negative impact of floods, the emergency services across Australia advise residents of flood prone areas to:


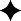
Make sure their home insurance or contents insurance covers flooding.


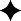
Look up relevant emergency phone numbers and placed them near or in their phones.


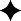

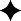
Prepare a home Emergency Plan for floods.

Prepare a home Emergency Kit for floods.

# Home Emergency Plan

- Select a suitable relocation destination for you and any other household members (including children and pets). Have all adult members of your household agree upon this.
- Identify what height floodwater can get to in and around your property (council/historical floods/SES) and at what heights local roads close. Determine what height this corresponds to on your local flood gauge (river) or what level of local rainfall contributes to (flash) flooding. Have all adult members of your household agree upon this.
- Have all adult members of your household discuss or consider how different water levels and other reasons for road closures might impact on evacuation/relocation plans.
- Identify alternative evacuation routes and determine whether and how such road closures would impact the triggers for evacuation (e.g., whether certain road closures may mean you need to leave earlier than anticipated).
- Have all adult members of your household think carefully about what (each of) you would need to do in response to a flood, and how this would be affected by different circumstances, including when different householders are at different locations (e.g., one is at home, one is at work, kids at school), or when certain householders may not have access to a vehicle (e.g., due to another householder using it and not being home) or at different times of the day/week (e.g., at night).
- You (and anyone who might be involved in the plan) need to take into account atypical or unexpected situations (e.g., certain family members not being at home even though they normally are, or friends/family visiting who are unable to evacuate themselves without help), and agree with all relevant people on who will do what in such situations.
- You need to be aware of both the flood warnings system that is relevant for your locality and of natural signs of flooding so that you can respond quickly.
- You need to identify specific triggers for starting to undertake preparatory actions and/or leaving (e.g., height on flood gauge, getting a Bureau of Meteorology (BoM) warning on a mobile phone, hearing a warning on radio) and have all adult members of your household agree upon these.
- List all important things to do and remember in case of a flood (written or typed on computer, phone, etc.).
- You (and your family/household) need to practice/rehearse your flood plan at least once over the last 12 months.
- Think about the consequences a flood would have on you and your household in the days, weeks, and months following the flood (e.g., your employment, schooling, etc.), and plan for these consequences to minimise their impact.
- All adult members of your household need to agree upon this plan.
- Ensure that all people who play a part in the successful execution of your flood plan (e.g., household members, neighbours, people you plan to stay with) are aware of the plan and have agreed to their role in the plan.
- You (and your family/household) are aware of your community’s flood plan.

# Emergency Kit

Ideally, an **Emergency Kit** for foods should contain the following items:


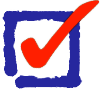

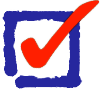
Portable battery-operated AM/FM radio with spare batteries Torch with spare batteries


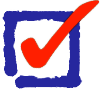
A folder with copies of important documents and records (e.g., insurance, passport, prescriptions)


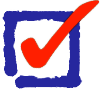

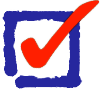
First aid kit and manual (with medical supplies necessary for your household) Waterproof bag/container for your valuables


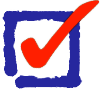
Candles and waterproof matches


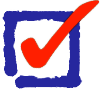
An emergency source of lighting and warmth/cooling that could be used in the days following a flood


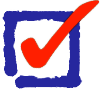
Sturdy gloves


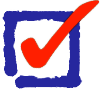
Long life food (3 days supply) including the ability to prepare the food in the days following a flood


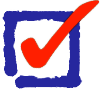
Bottled water (3 days supply at 3 litres per person per day)


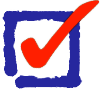
A waterproof bag/container for the above items to be placed in


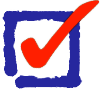
**Additional items**, ready to be put in the kit immediately when needed: Mobile phone and charger


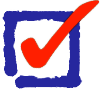
Prescription medicine


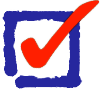
Pet needs


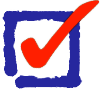
At least one pair of closed-toed shoes or boots for everyone in the household.


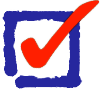
Clothes for every household member (3 days)

# Insurance Policy

To determine if your insurance policy covers floods, you will need to read the product disclosure statement that relates to your particular policy. Most likely your insurance company would have sent this to you, but often you can also find these disclosure statements online at the insurance company’s website or by performing an internet search for the name of your policy and the name of your insurance provider. If not, you will need to contact your insurance company directly.

# Emergency Contacts

If you are in **Victoria**, please follow the link below for a list of emergency contact numbers to put next to or in your phone: Emergency contact numbers for Victoria

If you are in **New South Wales**, please follow the link below for a list of emergency contact numbers to put next to or in your phone: Emergency contact numbers for New South Wales

***Please bookmark this page now so you will have the information when you need it.***
